# Transmural palliative care by means of teleconsultation: a window of opportunities and new restrictions

**DOI:** 10.1186/1472-6939-14-12

**Published:** 2013-03-07

**Authors:** Jelle van Gurp, Martine van Selm, Evert van Leeuwen, Jeroen Hasselaar

**Affiliations:** 1 Department of Anesthesiology, Pain and Palliative Care, Radboud University Nijmegen Medical Center, Geert Grooteplein 10, Nijmegen, GA 6525, Netherlands; 2 Amsterdam School of Communication Research, University of Amsterdam, Kloveniersburgwal 48, Amsterdam, CX 1012, Netherlands; 3 Department of Ethics, Philosophy and History of Medicine, Radboud University Nijmegen Medical Center, Geert Grooteplein 21, Nijmegen, EZ 6525, Netherlands

**Keywords:** Telecare, Audio-visual teleconsultations, Palliative care, Transmural care, Integrated health care, Mediation of professional-patient relations, Ethics, Philosophy of technology

## Abstract

**Background:**

Audio-visual teleconsultation is expected to help home-based palliative patients, hospital-based palliative care professionals, and family physicians to jointly design better, pro-active care. Consensual knowledge of the possibilities and limitations of teleconsultation in transmural palliative care is, however, largely lacking.

This paper aims at describing elements of both the physical workplace and the cultural-social context of the palliative care practice, which are imperative for the use of teleconsultation technologies.

**Methods:**

A semi-structured expert meeting and qualitative, open interviews were deployed to explore professionals’ assumptions and wishes, which are considered to contain latent presumptions about the practice’s physical workplace and latent elements of the cultural-social context, regarding (1) the mediating potential of audio-visual teleconsultation, (2) how the audio-visual teleconsultations will affect medical practice, and (3) the design and usage of the teleconsultation technology. We used a qualitative analysis to investigate how palliative care professionals interpret the teleconsultation package in preparation. The analysis entailed open and axial coding techniques developed in a grounded theory approach.

**Results:**

Respondents assume: 1. teleconsultation will hinder physical proximity, thereby compromising anamnesis and diagnosis of new or acutely ill patients as well as “real contact” with the person behind the patient; 2. teleconsultation will help patients becoming more of a pivotal figure in their own care trajectory; 3. they can use teleconsultation to keep a finger on the pulse; 4. teleconsultations have a healing effect of their own due to offered time and digital attention; 5. teleconsultation to open up an additional “gray” network outside the hierarchical structures of the established chain of transmural palliative care. This network could cause bypassing of caregivers and uncertainty about responsibilities; 6. teleconsultations lead to an extended flow of information which helps palliative care professionals to check the stories of patients and medical specialists.

**Conclusions:**

Professionals assume teleconsultation co-defines a new patient–professional relationship by extending hospital-based caregivers’ perceptions of as well as attention for their patients. At the cost, however, of clinical and personal connectedness. Secondly, a hermeneutics is needed to carefully interpret teleconsultation images. Thirdly, teleconsultations transform caregiving cultures as formerly separated care domains collide, demanding a redefinition of roles and responsibilities.

## Background

In their ambition to provide specialized care to palliative patients who desire to stay at home during the final stages of their illness, the staff of the Expertise Center for Palliative Care, Nijmegen – a Dutch, hospital-based, interdisciplinary center for comprehensive palliative care service [1] – decided to explore the possibilities of telecare. Telecare is defined as “technical devices and professional practices applied in ‘care at a distance’, care that supports chronically ill people living at home” [2].

Research about telecare for elderly people and for patients with chronic health conditions such as diabetes, heart disease, and chronic obstructive pulmonary disease inspired us to explore telecare within palliative care. In this research, corresponding elements of care such as independently “aging in place”, building and maintaining meaningful human interactions [3], symptom control, and “just-in-time preventive care” [4] proved feasible with telecare. Zooming in on synchronous audio-visual teleconsultations in the practice of palliative care at home, scarce literature offers some descriptions of the expectations of those involved. In two studies, a nurse experienced in palliative home care, a practicing oncologist, a hospice medical director, and a medical student researcher consider 40% [5] to 65% [6] of palliative care nursing visits potentially deliverable via video-visits. Further, representatives of American hospice programs and agencies (administrators, nursing supervisors, nurses, and social workers) see telecare offering patient assessments and quick responses to acute problems, provided that there is high-quality video. Telecare would relieve anxiety and contribute to patients’ and families’ peace of mind [7,8]. However, the first two studies [5,6] judge that crisis interventions at the request of the family cannot be replaced with video-visits. The same applies to visits including informal caregivers, medical interventions, or visits for psychosocial support care [5,6]. Moreover, the idea that video-visits should mainly function as an addition to already existing caregiving procedures instead of replacing them is also presented [7]. In conclusion, the literature about audio-visual teleconsultations in the practice of palliative care at home does not provide consensus about the application and value of teleconsultation. This lack of consensus compels us to look for more evidence about context-tuned telecare.

In line with Don Ihde’s considerations [9,10] about humans adopting new technologies, we think that teleconsultation technology is multistable: its eventual, stable appearance and function depends on how participants of a particular practice, and in this case, the palliative care practice, adopt this technology in both their particular physical workplace (e.g., Internet connections and rooms for private conversations) and in their particular cultural-social context, i.e., their collective normative frameworks and daily routines [2,10]. Whether and how they do this, and whether and how they allow the technology to change their existing practice, will eventually define the fit of the technology [10].

The purpose of this study was to describe these elements of the physical workplace and the cultural-social context of the palliative care practice, both of which are imperative to implementing a teleconsultation technology. We therefore analyzed interviews with professionals (respondents) playing differing roles in our hospital-based expertise center for palliative care. Each respondent has his/her own expert perspective on the future implementation of teleconsultation in daily practice. At the core of these interviews are the respondents’ initial assumptions about the intermediate position of teleconsultation technology in caregiving as well as their wishes about its design and function. These assumptions and wishes contain latent presumptions about the practice’s physical workplace and latent elements of the cultural-social context: real and relevant presuppositions that define the implementation of teleconsultation. In the final section of this paper, we will use, among others, the phenomenology of human-technology relationships [9,10] to broaden our research perspective for an extended follow-up study of patients’, general practitioners’, and hospital-based palliative care professionals’ experiences with teleconsultation in the practice of palliative care at home.

We address the following research questions:

1. What do professionals in a Dutch, interdisciplinary, palliative care center assume about the mediating potential of audio-visual teleconsultation before its implementation?

2. What do they assume about how the audio-visual teleconsultations will affect their current medical practice?

3. What features do they want to be included in the design and application of the teleconsultation technology?

## Methods

This study uses qualitative methods based on Glaser and Strauss’ grounded theory [11,12]. A semi-structured expert meeting and qualitative, open interviews with professionals in the hospital-based Expertise Center for Palliative Care were deployed in order to discover the existing assumptions and wishes about teleconsultation in palliative care. We purposefully sampled respondents from those who are responsible for implementing teleconsultation in their daily working routines. All five respondents were experts in palliative care with different professional roles and responsibilities (Table 1).We used qualitative analysis, entailing open and axial coding techniques, to investigate the existing assumptions and desires. We requested the approval of the ethics committee, but this was not required because no patients were involved at this stage of research.

**Table 1:** Respondent characteristics

**Expert meeting respondents**	**Interviewees**
*1. Staff/palliative care physician*: the Head of the Expertise Center for Palliative Care at Radboud University Nijmegen Medical Centre; an anesthesiologist–pain physician	*1. Palliative care physician*: specialist in medical oncology
	*2. Nurse practitioner et cetera*	*2. Nurse practitioner palliative care*: intended teleconsultant.
*3. Staff*: the department’s policymaker and assistant professor for health care innovations, who coordinates the teleconsultation project		
*4. Palliative care physician*: specialist in elderly care		

### Data collection 1 - the expert meeting

In the expert meeting, we discussed the palliative care team’s collective normative frameworks, their daily routines, and the potential impact of teleconsultation on their daily work with two physicians and a member of staff. The principal investigator (JG) was panel chairman during the meeting.

We developed a discussion guide on the basis of an operationalization of the “current medical practice of the palliative care center” (RQ2). The guide contained four topics for discussion: (1) initial contact with a patient, (2) taking care of a patient in need, (3) interacting with family physicians and patients, and (4) being responsive to the impending death of a patient. A hypothetical case introduced each topic in a (Table 2). We asked those present to discuss these hypothetical cases. The principal investigator used clarifying and probing questions when elements of the discussion were unclear, particularly when the discussion touched on mediation of interactions by the future teleconsultation technology. The expert meeting took 1 hour. The discussions were audio-recorded, and the recordings were transcribed verbatim into a 15-page transcript.

**Table 2:** The expert meeting’s topics of discussion with a short description of their hypothetical cases


**1. Initial contact with a patient**	Patient X has now received teleconsultation equipment. He is, so far, unknown to the palliative care team. How should a care trajectory with this patient start? Face to face in real life or on a screen?
*Referring to RQ1, 2*	
**2. Taking care of a patient in need**	Patient Y has been in the research project for a few weeks now. Her condition is rapidly deteriorating. During a teleconsultation, the nurse practitioner of the palliative care team is confronted with an urgent situation. What should she do? Should patients be able to initiate contact with the palliative care team via teleconsultation?
*RQ2*	
**3. Interacting with physicians *and *patients**	The family physician, who is the primarily responsible caregiver, decides to ignore the advice of the palliative care team. He pursues his own treatment plan with the patient. This becomes clear to the palliative care team during the next teleconsultation. How should the team react? What if the family physician and the palliative care team insist on different treatment policies?
*RQ2*	
**4. Being responsive to the impending death of a patient**	Patient Z is about to die. The palliative care team has had intensive contact with her and her family via teleconsultations during the past 3 months. How should they close this care trajectory?
*RQ1, 2*	

### Data collection 2 - the interviews

The interviews, following the expert meeting, focused on personal assumptions and wishes of the palliative care team members who were about to do their caregiving work with the support of teleconsultation. The principal investigator (JG) conducted three intensive, in-depth interviews (45–70 minutes), one with the nurse practitioner and two with the palliative care physician specialized in medical oncology. JG based his initial interview questions on the hypothetical cases; clarifying and probing questions followed closely, asking who, how, why, etc. (Table 3). The tentative teleconsultation protocol described in the following paragraph was also used as a trigger for conversation during the interviews. All interviews were audio-recorded, and later transcribed, partly verbatim, to 13 pages of condensed transcript.

**Table 3:** Two examples of interview topics with initial questions


**1. Initial contact with a patient**	Patient X has received teleconsultation equipment. He is still unknown to you. How should you start a care trajectory with him? Face to face in real life or on a screen?

	Example of a probing question: What makes you think so?

**2. Being responsive to the impending death of a patient**	Patient Y is about to die. You have had intensive contact with her and her family via video consultations during the last 3 months. How can you close this care trajectory appropriately?

### Tentative protocol for synchronous, audio-visual teleconsultation in home-based palliative care

**Figure 1:**
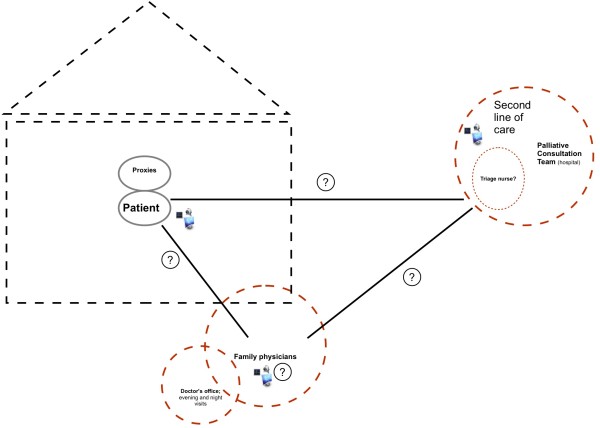
Tentative teleconsultation protocol for the practice of palliative care.

The Expertise Center for Palliative Care of the Radboud University Nijmegen Medical Centre currently explores, within a transmural research project, synchronous, audio-visual teleconsultation via webcam and iPad. The initial research proposal contained a tentative teleconsultation protocol structuring the collaboration between a hospital-based palliative care center, family physicians, and home-based patients (Figure 1). The general aim of this telecare-supported collaboration was, and still is, to jointly design pro-active palliative care by combining the expert knowledge of the hospital-based palliative care specialist, stimulated by the audio-visual conversations with home-based patients via the teleconsultation technology, with the family physician’s contextual knowledge of the patient, as well as the patient’s expertise of his/her own disease.

The hospital-based palliative care team consists of one nurse practitioner, two nurses, and five palliative care physicians who carry out the protocol in different line-ups: one nurse practitioner or nurse makes inventories with the patient of his/her symptoms and other problems once a week via video conversations, while a palliative care physician monitors in the background. The patient’s family physician is expected to be involved either by joining the video conversations at the patient’s home or afterwards when the nurse practitioner or palliative care physician discuss their findings with the family physician.

### Analysis

In line with the guidelines for analysis based on the Grounded theory approach [11,12], the analysis started with open coding [11], also called “initial coding”[12]. Sticking closely to the data [12], the principal investigator (JG) ascribed conceptual labels to the text to “describe the essence of what is being expressed” by the respondents [11]. We used CAQDAS Atlas.Ti to systemize the analysis.

In the second step of the analysis, we applied a two-fold “constant comparative analysis” [11]. First, we used two salient elements, *mediation* (RQ1) and *interactions in the current medical practice of palliative care* (RQ1/2), to broadly categorize the available codes. Second, within the boundaries of these broad categories, JG constantly compared incidents and accompanying codes to define more specific, substantive categories. He presented these categories to the research team as part of peer debriefing. During these comparisons, the research questions were at the basis of the analytic gaze as to distinguish (a) the respondents’ definition of their current practice of palliative care and (b) the respondents’ assumptions about how teleconsultation would affect their own medical practice and the practice of palliative care in general. We labeled the newly found categories with concepts that were elaborated on in concept-indicator models.

**Figure 2:**
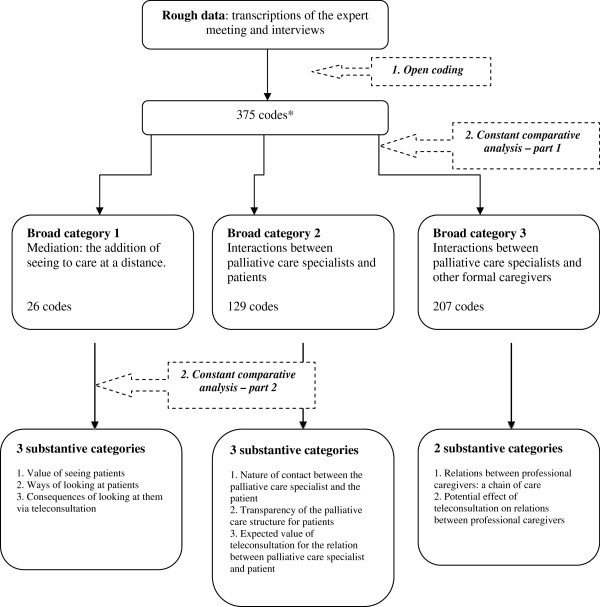
**Schematic overview of the analysis.** *14 codes proved irrelevant for answering the research questions and were not classified in one of the broad categories.

In the third step of the analysis, we focused on similarities and differences when we specified relationships between categories. Schematic overviews described the definition of the practice of palliative care and the assumptions about how teleconsultation would affect medical practice as seen from the perspectives of the physicians, member of staff, and the nurse practitioner. We created schematic overviews to clarify the relationship between (a) the definition of the current practice of palliative care, (b) teleconsultation, and (c) presumptive future practice of palliative care including teleconsultation. Figure 2 gives a schematic overview of the analysis.

We took three measures to ensure validity during our analysis, i.e., the extent to which the categories correctly represent the phenomenon under study (concept validity) and the extent to which the applied procedures provide access to the phenomenon under study (internal validity) [13]. First, as part of the peer debriefing [14], co-authors MS, JH, and EL repeatedly discussed the coding and categorizing with JG. Second, the interviewer’s frequent summaries of respondents’ stories offered the respondents a chance to correct their stories immediately. As part of the same respondent validation, the Head of the Expertise Center for Palliative Care scrutinized the categories and their mutual relationships after the analysis. Third, we interpreted the findings in the context of current theoretical frameworks (see Discussion).

## Results

In response to the first research question about the mediating potential of audio-visual teleconsultation, the respondents started with describing three types of connectedness they experience with their patients. Teleconsultations are supposed to have an impact on each of these types of connectedness (Table 4).

**Table 4:** An overview of the expected mediation of interactions between patient and palliative care professionals by teleconsultation

	**Expected value of teleconsultation for the professional–patient relationship**			
**Types of connectedness between patients and professionals**	1. Teleconsultation **complicates connectedness** with patients	2. Teleconsultation **redefines the position of the home-based patient** for better (being in control) or for worse (bringing the hospital into the home)	3. Teleconsultation might be **an enrichment**: keeping a finger on the pulse	4. Teleconsultation has **a healing effect**.
1. **Keeping in touch**	Respondent: “After I’ve seen the patient once, the following contacts could be virtual. But I want to look a patient in the eye once. What one calls ‘smelled, felt, and seen”	Respondent: “Well, you have to consider that demolishing barriers in favor of patients, by means of quick access and good, because direct, sight…, also has the disadvantages of [the physician] being directly exposed, a frustrated chain of care, and potential hospitalization [of the patient].”		Respondent: “Contact alone has a healing effect.”
2. **Clinical hands**	Respondent: “But a physical examination is impossible, as you cannot work through the screen (laughs).”		Respondent: “…[to prevent] patients arriving at the hospital about whom I think: ‘You should have kept him at home, family physician, because this patient is already dying.”	
3. **Seeing the person behind the patient**	Respondent: “For me [teleconsultation] still doesn’t feel like having real contact with a patient.”	Respondent: “Or the patient needs it [an initial face-to-face meeting]. That is also quite possible, since I am a stranger to these patients.”		

### Mediation in teleconsultation: interactions between palliative care professionals and their patients

#### Three types of connectedness

The respondents report that they initiate regular, brief, and superficial conversations, most often by telephone, in order “to keep in touch with a patient”. These conversations make professionals feel involved in the ongoing care for their patients. It also assures them that they are indeed always accessible for their patients. However, such brief, superficial contacts do not suffice for new patients or for cases of acute complications of any patient. The professionals emphasized, during the expert meeting and interviews, that they want to be near the patient in such cases to examine the bodily symptoms. A second type of connectedness between professional and patient then emerges: the patient becomes subjected to direct, clinical hands contributing to good anamnesis and diagnosis, also referred to as “normal medical care”. From this point on, palliative care professionals try to deepen the relationship with their patients, as they go beyond their clinical examination in search of “real contact” with the person behind the patient. They describe a gradual grading of their clinical examination of patients into a multidimensional approach, the third type of connectedness, where physical, psychological, social, and spiritual suffering are taken into account. They gain a valuable “gut feeling” from this holistic, all-encompassing connectedness, usually referred to by palliative care professionals with the rather down-to-earth phrase “to see, smell, and touch patients”. Holistic connectedness is at the base of “integrated medical care”.

#### Teleconsultation: complicating connectedness with patients

We found that respondents implicitly assume that face-to-face interaction is at the basis of both clinical and holistic multidimensional care. Palliative care professionals expect a teleconsultation application to hinder both kinds of care because the loss of physical proximity compromises clinical examination and getting acquainted with the person behind the patient. Without physical proximity, palliative care professionals know they cannot touch or smell a patient, but have to rely on sight and hearing alone. They also worry that they cannot truly touch upon patients’ personalities, realms of thought, and social worlds. As a consequence, palliative care professionals feel that, with teleconsultation, they would lose meaningful tools for adequate diagnosing.

Moreover, the audio-visual information that teleconsultation can provide should, according to one respondent, be viewed with scepticism, as “the directness and ease with which we see could lead to the misconception that seeing [alone] is understanding”. This respondent emphasizes that the sight of a patient can only be valuable for diagnosing if the visual information is authentic and an integrated part of clinical reasoning, in which a variety of perceptible information is used to either verify or discredit certain interpretations.

#### Teleconsultations redefine the patient’s position in the palliative care model

The respondents said that teleconsultation could make them easily accessible to their patients. They expect patients to become pivotal figures who have quick, easy, and personal contact with their caregivers. They are, however, ambivalent about the unlimited access that teleconsultation gives patients. Besides having the aforementioned up side of accessibility, professional caregivers see a down side to teleconsultation, which lowers thresholds too much and increases their workload. Palliative care physicians also fear increasing dependency of patients due to being too accessible: by potentially dismantling a vertical care model with clearly defined, consecutive phases of referrals from general to expert care, the patient’s unrestrained searching for both information and help is encouraged. This would diminish patients’ abilities to solve some of their own problems and pave the way for “hospitalization at home”.

#### Keeping a finger on the pulse

The respondents said teleconsultations are useful when deployed as a tool for keeping a distant eye on the patient. They are supposed to help them to continuously determine the patient’s state and to make well-informed decisions in case of observable calamities. However, this monitoring of a patient can only be applied after the palliative care professional has seen the patient face to face first and has gained some sense of the patient and his/her care demands. One respondent expected an initial face-to-face contact to be a basic necessity for the palliative care professionals and for the patient because both will likely have the same desire to reduce a sense of being strangers to each other, particularly in the last phase of life.

#### The healing effect of teleconsultation

Palliative care professionals think that teleconsultations have a healing effect of their own. The extra contact and attention are believed to be helpful for patients: the teleconsultation application and teleconsultations are perhaps not considered to heal the patient in a strict medical sense, but they could generate a feeling of being well taken care of.

### Mediation by teleconsultation: interactions between palliative care professionals and other medical professionals

In response to the second research question regarding how the audio-visual teleconsultations will affect their current medical practice, the respondents expect the established chain of care to be vulnerable to unwanted changes due to teleconsultation (Table 5).

**Table 5:** An overview of the mediation of interactions between palliative care professionals and other medical professionals by teleconsultation

	**Expected value of teleconsultation for the relationship of one palliative care professional to another medical professional**	
**Established chain of palliative care: relationships between diverse caregivers**	Teleconsultations as a means **of bypassing hierarchical structures**	Teleconsultations as a means **of gaining access to the home**
1. **The autonomous director of the play**	Respondent: “…if patients have open access, if they push a button and the caregiver of their choice directly appears on the screen, then the slackening aspects of the chain of care, which could help restore its normal course, are gone… Right now, the chain of care is ignored in several ways, with the up side of short cuts and the down side of people being passed over, of people who no longer know what’s going on or act on the basis of old information. But there’s still a certain barrier nowadays.”	Respondent: “…the family physician treats the patient [residing at home]. And we have to control our tendency to completely take over from the family physician, because that’s not the way it is supposed to be.”
2. **Dependent on other caregivers’ stories**		Respondent: “If you look for yourself, you see a patient totally different from the one you’ve been told about on the phone [by family physicians]”

#### Teleconsultation facilitates bypassing hierarchical structures in the established chain of care

The respondents describe transmural palliative care as a chain of care consisting of hospital-based palliative care physicians and nurses, family physicians, and home care, each relying on an established hierarchy and a shared expectations about responsibility. If the patient stays at home, the family physician is the “physician in charge”. He/she is expected to be the director of the patient’s care and to actively attune care with both the patient and other caregivers.

If the physician in charge, for whatever reason, decides to refer this patient to hospital-based palliative care professionals, the latter have an obligation to see the patient to be able to take the responsibility handed to them. They consider teleconsultation a helpful instrument with which they can fulfill this obligation to see the patient immediately. However, some believe that adding teleconsultation to the practice of transmural care breaks open the previously well-defined domains of the hospital and the home: a ‘gray’ network will emerge in which responsibilities are no longer clearly defined in advance. The respondents acknowledge that it is not unusual in the practice of palliative care to bypass responsible caregivers for reasons of efficiency or due to disagreements, but they feel that teleconsultation would facilitate this bypassing and open up the domain of transmural care in a new way. A hospital-based palliative care professional could, for example, see a patient vis-à-vis on a screen without permission from the physician in charge. This would instantly change the relationship between the caregivers: after seeing the patient, the professional can no longer remain an indifferent advisor to the physician in charge and becomes fully involved, bearing responsibility for the patient in virtual face-to-face contact. Other foreseeable possibilities include patients residing at home who use teleconsultation to bypass their family physician in favor of the hospital-based professional, or family physicians bypassing a certain professional, e.g., an oncologist, to deal directly with the palliative care physician.

If teleconsultation results in bypassing responsible caregivers, this would, said one respondent, directly endanger an advantage of the well-functioning chain of care: its ability to slow things down. A sluggish chain of care is believed to provide professional caregivers with more time to collect fragmented information and to become better informed. Moreover, as patients have to engage several formal caregivers before reaching their goals, there is a good chance that shortcomings in treatment in an earlier stage will be compensated along the way.

#### Teleconsultation as a means of gaining access to the home

The respondents view teleconsultation as a potential tool for accessing the home, normally the domain of the family physician. Having audio-visual access to the home likely means getting information they usually lack, such as the looks of a patient and his/her household. Information with which they can check the stories of patients and physicians in charge. The respondents sometimes question these original stories: they realize that their own observations might differ from those of their colleagues; their own, they say, are usually more nuanced. Of course, this questioning is variable as palliative care professionals maintain good relations with some family physicians or medical specialists, with whom they communicate easily, while mutual understanding with others is almost certainly ruled out. The newly acquired information is supposed to be useful for convincing physicians in charge to adopt an adapted treatment plan. Although the hospital-based professionals sense an internal inclination to take over the physician in charge’s work, they show awareness that, especially in case of teleconsultations, they have to maintain self-control to keep the established chain of care intact.

### An extended teleconsultation protocol

**Figure 3:**
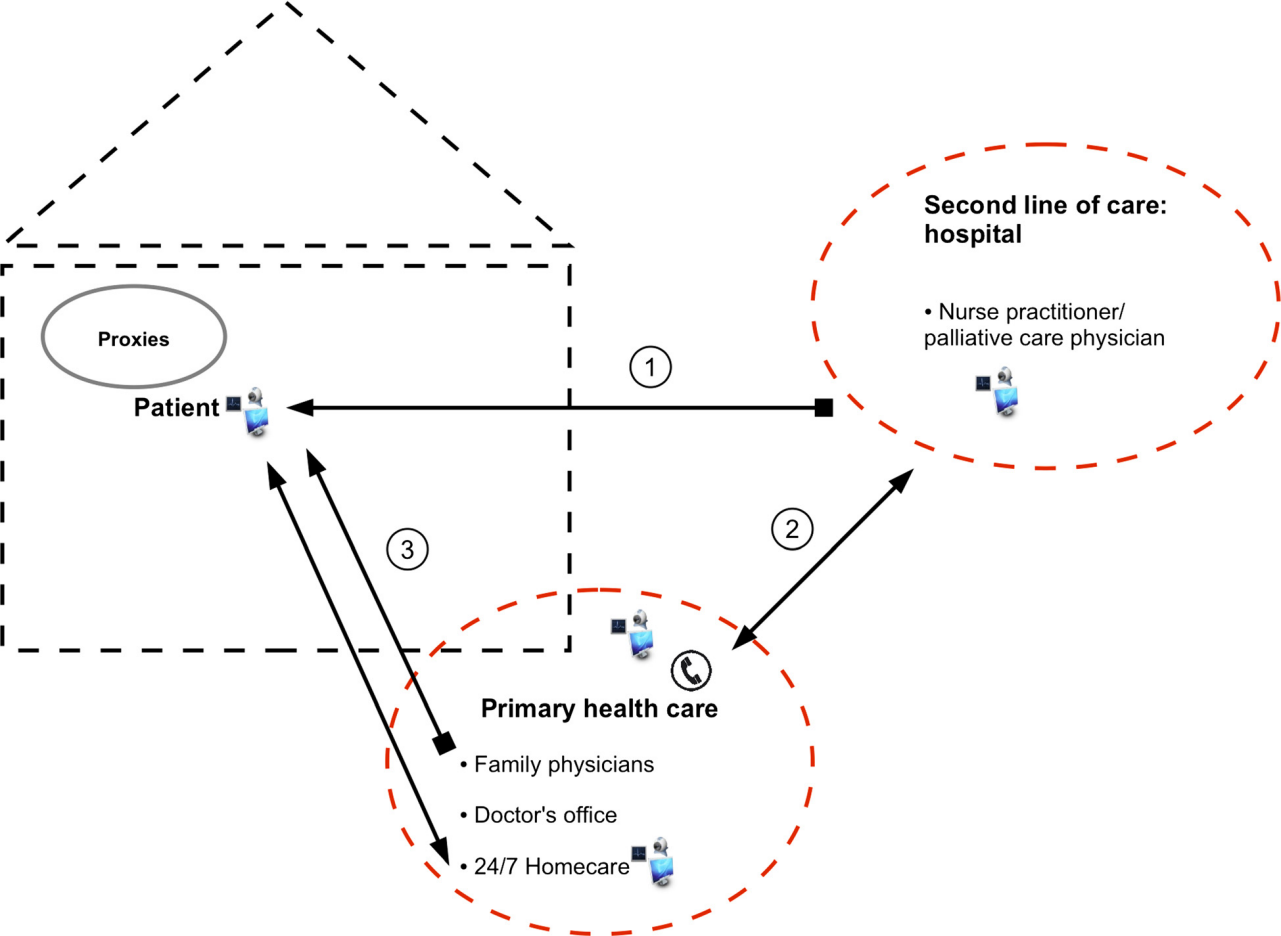
Extended teleconsultation protocol for the practice of palliative care.

In response to the third research question about their wishes for the design and application of the teleconsultation technology, the respondents collectively came up with an extended teleconsultation protocol (Figure 3). This protocol was used at the start of the implementation:

1. In advance, the home-based palliative care team insists to see each patient face to face at least once, before the patient gets enrolled in a teleconsultation trajectory.

1. The nurse practitioner initiates the teleconsultation with a patient at a set time during working hours. The patient cannot contact the hospital-based palliative care team via the teleconsultation route, which prevents bypassing in the chain of care. The nurse practitioner makes an inventory with the patient of his/her symptoms and other problems, but does not make treatment decisions. The nurse practitioner can provide practical nursing advice.

2. The nurse practitioner reports her findings to the palliative care physician and to the family physician (in case the family physician was absent from the patient’s home during the teleconsultation). Further medical treatment and care are discussed with the family physician. This discussion is documented in an electronic patient file.

3. The family physician decides, ideally with the patient, about further treatment and care. This treatment plan is communicated to the family physician’s office and the home care institution.

## Discussion

We have found that the interviewees presume they can use teleconsultation to keep a finger on the pulse of patients residing at home, provided that at least one initial face-to-face contact precedes the teleconsultations. Furthermore, if responsibility for a patient is handed over to palliative care professionals, teleconsultations are supposed to fulfill the professional’s need to see this patient immediately. Teleconsultations are even presumed to have a healing effect of their own, as time and digital attention are offered to patients. The members expect that teleconsultation will facilitate more patient-centered care. These advantages may go hand in hand with limitations: the respondents working in the expert center conjecture that teleconsultation will complicate the connectedness with their patients: the physical proximity, and thereby the quality of diagnostics is supposed to become more difficult due to teleconsultations. “Real contact” with the person behind the patient could never be realized by means of teleconsultations alone. Besides, the increased, virtual availability is believed to facilitate patients’ sometimes unrestrained and uncontrolled queries, which may cause increasing dependency and heavy workloads. Teleconsultation is expected to open up an additional ‘gray’ network outside the hierarchical structures of the established chain of transmural palliative care. This network could make it possible to bypass caregivers in charge, causing uncertainty about responsibilities. Medical specialists, family physicians, and nurse practitioners could then easily depart from the treatment policy for a patient either at the hospital or in the home, possibly creating confusion and miscommunication.

As mentioned in the introduction, teleconsultation technology is considered multistable [9,10]. Teleconsultation involves a technical computer device as well as a designed care process due to the introduction of this device. The fit of this computer device in palliative caregiving is defined by its embeddedness in the particular physical and cultural-social context of the caregiving practice. In this sense, teleconsultation is “a hybrid affair” [15] as it only appears as a compilation of human action and teleconsultation technologies: the latter encompasses a script that frames social action, roles and identities, but end-users will co-design the telecare services as well [16,17]. This article’s teleconsultation protocol is a first attempt of hospital-based professionals to condition the offered teleconsultation technology to their daily practice. It is the end-point of this paper, an accumulation of professionals’ assumptions on extended teleconsultation into a palliative telecare service. In future studies, we will investigate more closely whether and how the hospital-based professionals are able to manage both the technology and the service according protocol, as well as whether and how other end-users, like patients, families and family physicians, co-design this palliative telecare product.

In the following, we elaborate on a few theoretical notions on the possible teleconsultation technology-user relationships that could sensitize us to meaningful events for future studies.

### Perceiving a patient via teleconsultation: the embodiment relationship

Teleconsultation technology facilitates *experiencing through technology*[9] for both patients and caregivers. This kind of mediation will extend certain bodily capacities and neutralize others, thereby magnifying certain aspects of the observed world and reducing others. The respondents referred to this embodiment-relationship: they expect extended teleconsultation technology to extend hospital-based professionals’ perceptions of patients, who can now be seen in their homes. Furthermore, continuous, interpersonal, digital contact between patients and caregivers might magnify a patient’s healing capacity [18].

Further research should address the following questions: how do caregivers actually improve their care when audio-visually communicating with the patient at home? What will be the nature of the mutual digital contacts between specialists and patients? What do teleconsultations exactly mediate that contributes to healing effects?

With regard to the magnification/reduction structure [9], Ihde claims that, normally, “what is revealed [by technology] excites, and what is concealed may be forgotten”. It is not so much the magnification as the reduction that stands out for the interviewed palliative care professionals: for them, teleconsultation technology compromises connectedness with the patient in that it does not create physical proximity and diminishes professionals’ capacity to get in touch with the person behind the patient and develop a “gut feeling*”*. This fear seems, at least partly, pertinent as heart-failure nurses working with telemonitoring actually experience losing intimate knowledge of the patient and his/her psycho-social well-being by losing physical proximity [19]. However, interpersonal bonds in modern workplaces, where “telecommuting” is part of daily practice, depend the least on physical proximity [20].

This foreseen reduction raises questions: how does the loss of physical proximity frustrate adequate diagnosing and what does this mean for palliative care at a distance [21,22]? How can sympathizing and empathizing with patients be maintained in teleconsultation, and how do patients experience this new proximity? Moreover, researchers should investigate the added value of telecare consultation after initial face-to-face contact [5,6,18].

### Looking at a screen: the hermeneutic relationship

Besides being something *through* which caregivers get in touch with their patients, the teleconsultation technology is also an instrument palliative care professionals *look at* to interpret the patient’s status. The professionals have to learn how to interpret their patients’ images and stories to apply them in a responsible way in medical practice [9]. Interpreting an image of the home is part of the palliative care physician’s diagnosis and has to be learned, similarly to a gynecologist needing to learn how to interpret a sonogram and a radiologist how to interpret magnetic resonance images [23]. An extended follow-up study must include questions about the different interpretative frameworks the communicators use. What do patients and professionals look for in the images? How do professionals and patients know if the teleconsultation images and sounds, in a “semi-opaque cooperation with referent objects” [9], truthfully refer to the appearance of the conversation partner and his/her social context [24]? Methods of truth-finding in a virtual context should be part of a follow-up study. We caught a glimpse of such a method when one respondent argued for scepticism towards the teleconsultation images: they can only be diagnostically valuable if verified by clinical reasoning combined with a variety of other information, such as patients’ or colleagues’ stories or lab results; to see a patient with yellow skin simply does not suffice for building a diagnosis.

### Transforming transmural care and transporting culture

Teleconsultation in palliative care never fulfills a neutral role [9,10], but co-shapes the experiences and perceptions of the surrounding world. Teleconsultation is thought to give patients a feeling of control over their care. However, the respondents think this patient-centeredness comes at a cost: teleconsultations might upset the balance of involvement and professional distance, leading to loss of control over “boundaries between absence and presence” [25]. A shared freedom to initiate teleconversations is likely to transform expectations and obligations between professionals and patients [22,26]: when patients expect continuous access, professionals feel more obliged to be present and to respond quickly and accurately. It is a feeling that goes beyond their earlier advisory role.

Another result of the same shift to quick, easy, and more reciprocal audio-visual communication between patients and professionals is the blending of different domains (home and hospital) into one still developing transmural area. Profound relationships can develop between patients and caregivers who were formerly restricted to a particular domain. These more intense relationships might contribute to a mutual trust and to a patient’s or informal caregiver’s peace of mind [8], or to mutual dependency at a distance. The feelings of trust and/or dependency could tempt patients and professionals alike to bypass less involved caregivers. This would subvert the hierarchical structure of the established chain of care and create a void when it comes to taking responsibilities.

The teleconsultation equipment itself also functions as a medium for *transporting* cultures. It is presumed to create a more pluricultural environment [9] in home and hospital. The hospital culture may intrude in the home through the technology. Patients and family physicians might also bring their formerly home-bound cultures into the hospital, although the hospital-based respondents did not mention this. These interactions of cultures might have consequences for the use of life-prolonging treatment, terminal hospital admissions, and multidimensional decision-making at life’s end. This requires future research.

### Limitations

Although valid, our results originate from a study with a limited number of respondents. Despite the small sample, this case study provides valuable insights into the cultural-social context of palliative care. The respondents are typical representatives of a palliative care practice, who prefer to work within integrated care processes. Most respondents were experienced and older – not uncommon in a field where caregivers are constantly confronted with death and dying. Being experienced and older may have influenced them regarding new technologies. Moreover, they might fear technology more because, at first sight, it endangers certain types of connectedness that are essential parts of their holistic, humanistic care. Nonetheless, age has the advantage of being more independent of hierarchical structures. This creates freedom to participate in innovative projects.

Overall, this paper on the cultural-social context of the practice of palliative care in relation to teleconsultation is a solid base for further research into:

•Technology-mediated interactions between palliative care professionals and patients. The potential “disagreement [on], negotiation [on], and potential breakdown [of]” [16] this innovation will actually enhance access to this field of research as friction exposes itself more easily.

•The experiences of designers, palliative care professionals, patients, families, informal caregivers, and family physicians, all involved in working with teleconsultation within transmural palliative care. It is notable that our respondents only referred to teleconsultation technology fitting the professional cultural-social context. It is worth investigating whether and how the technology fits into the actual workplace and home.

•The mediation of professional, patient, and family relationships by teleconsultation.

•The moral assessment of teleconsultation in palliative care with regard to a dignified last phase of life.

With the results of such a follow-up study, teleconsultation structure can be designed for caregiving that benefits both patients and caregivers and fits the managerial, regulatory, and financial frameworks of the practice of transmural palliative care.

## Conclusion

For professionals residing in an expert center for palliative care, teleconsultation technology opens up a window of opportunities. It presumably helps patients establish themselves at the center of their own care, helps palliative care professionals see their patients at home and continue their care by keeping a finger on the pulse, and even heal at a distance. However, with new opportunities come new restrictions: teleconsultation technology does not allow for physical proximity, makes it more difficult to get a feel for a patient, and might disrupt the chain of care and the autonomy of the physicians in charge.

## Abbreviations

RQ: Stands for ‘research question’ (page 3 and further).

## Competing interests

The authors declare that they have no competing interests.

## Authors’ contributions

JG functioned as chairman of the expert meeting and conducted the interviews, carried out the qualitative analysis and drafted the manuscript. MS, EL, and JH have made substantial contributions to the analysis and interpretation of the data. All authors read and approved the final manuscript" to "All authors have been involved in revising drafts and read and approved the inal manuscript.

## Pre-publication history

The pre-publication history for this paper can be accessed here:

http://www.biomedcentral.com/1472-6939/14/12/prepub
